# Genes Involved in Maintaining Mitochondrial Membrane Potential Upon Electron Transport Chain Disruption

**DOI:** 10.3389/fcell.2022.781558

**Published:** 2022-02-16

**Authors:** Karthik Vasan, Matt Clutter, Sara Fernandez Dunne, Mariam D. George, Chi-Hao Luan, Navdeep S. Chandel, Inmaculada Martínez-Reyes

**Affiliations:** ^1^ Department of Medicine, Northwestern University Feinberg School of Medicine, Chicago, IL, United States; ^2^ High Throughput Analysis Laboratory and Department of Molecular Biosciences, Northwestern University, Chicago, IL, United States; ^3^ Department of Biochemistry and Molecular Genetics, Northwestern University Feinberg School of Medicine, Chicago, IL, United States

**Keywords:** mitochondria, CRISPR screen, mitochondrial membrane potential, ATP synthase, cell death, mitochondrial protein translation

## Abstract

Mitochondria are biosynthetic, bioenergetic, and signaling organelles with a critical role in cellular physiology. Dysfunctional mitochondria are associated with aging and underlie the cause of a wide range of diseases, from neurodegeneration to cancer. Through signaling, mitochondria regulate diverse biological outcomes. The maintenance of the mitochondrial membrane potential, for instance, is essential for proliferation, the release of mitochondrial reactive oxygen species, and oxygen sensing. The loss of mitochondrial membrane potential triggers pathways to clear damaged mitochondria and often results in cell death. In this study, we conducted a genome-wide positive selection CRISPR screen using a combination of mitochondrial inhibitors to uncover genes involved in sustaining a mitochondrial membrane potential, and therefore avoid cell death when the electron transport chain is impaired. Our screen identified genes involved in mitochondrial protein translation and ATP synthesis as essential for the induction of cell death when cells lose their mitochondrial membrane potential. This report intends to provide potential targets for the treatment of diseases associated with mitochondrial dysfunction.

## Introduction

Mitochondria are essential for cellular physiology. Defects in mitochondrial function are associated with aging and underlie the cause of a wide variety of diseases ranging from neurodegeneration to cancer (Hagen et al., 1997; Wallace, 2005; Desdín-Micó et al., 2020). Placed at the core of cellular metabolism, mitochondria have key functions as biosynthetic and bioenergetics organelles. Flux through the tricarboxylic acid cycle (TCA) generates metabolites that feed into anabolic pathways including nucleotide, lipid and amino acid synthesis (DeBerardinis and Chandel, 2016; Zong et al., 2016). For example, oxaloacetate and citrate are exported from the mitochondrial matrix to the cytosol to generate aspartate and acetyl-CoA for nucleotide and fatty acid synthesis, respectively. The TCA cycle also produces the reducing equivalents NADH and FADH2, that deliver their electrons to complex I (NADH dehydrogenase) and complex II (SDH) of the electron transport chain (ETC), respectively. Complexes I and II further transfer their electrons to the mobile electron carrier ubiquinone (CoQ) resulting in the generation of ubiquinol and the regeneration of the NAD+ and FAD cofactors, which allows oxidative TCA cycle to function. Ubiquinone can also receive electrons from dihydroorotate dehydrogenase (DHODH), an essential mitochondrial enzyme necessary for *de novo* pyrimidine synthesis. Other pathways, like fatty-acid and amino-acid oxidation, choline oxidation, and glycolysis, can also donate electrons to ubiquinone. Through the Q cycle, complex III accepts two electrons from ubiquinol and funnels them to cytochrome c. The respiratory chain ends with the reduction of the final acceptor, oxygen, to water by complex IV. The transfer of electrons through the ETC is coupled to the pumping of hydrogen ions from the matrix to the intermembrane space by complexes I, III and IV. This generates a proton gradient or proton motive force (PMF), composed of a small chemical (DpH) and large electrical membrane potential (DΨ), across the inner mitochondrial membrane, that is used by complex V (ATP synthase) to generate ATP through oxidative phosphorylation (OXPHOS).

Beyond their canonical metabolic functions, mitochondria also serve as signaling organelles, which accounts for their ability to regulate a wide range of biological outcomes, including proliferation, differentiation, and adaptation to stress (Chandel, 2014). Mitochondrial control of Ca^2+^-dependent signaling cascades is a key determinant of cell fate (Rizzuto et al., 2012; Raffaello et al., 2016). Some other examples of mitochondrial dependent cellular networks include the release of cytochrome c to induce apoptotic cell death, the activation of AMP-activated protein kinase (AMPK) under energy stress to control mitochondrial dynamics, and the release of mitochondrial DNA (mtDNA) to activate immune responses (Liu et al., 1996; West and Shadel, 2017; Herzig and Shaw, 2018). In addition, TCA cycle metabolites like succinate, fumarate or acetyl-CoA can control the expression of specific genes by diverse mechanisms, including the regulation of chromatin and DNA modifications (Ryan et al., 2019; Martínez-Reyes and Chandel, 2020). Finally, mitochondrial reactive oxygen species (mROS) are well-appreciated signaling molecules that can reversibly oxidize certain thiols within proteins to modify their function and have been implicated in the regulation of the hypoxic response through the stabilization of the HIF transcription factor (Chandel et al., 1998). Mitochondrial dependent ROS production is involved in physiological and pathological outcomes, such as immunity, cancer, and aging (Shadel and Horvath, 2015; Kong and Chandel, 2018).

Importantly, in our previous studies, by using genetic tools that allowed us to uncouple different respiration-linked mitochondrial processes, we uncovered that the maintenance of the mitochondrial membrane potential is essential for proliferation, the release of mROS, and oxygen sensing (Martínez-Reyes et al., 2016). Other key functions dependent on the mitochondrial membrane potential include solute and ion transport across the inner membrane, protein import of nuclear DNA-encoded proteins into the mitochondria, and the regeneration of mitochondrial NADPH by nicotinamide nucleotide transhydrogenase (NNT) for ROS homeostasis (Rydström, 2006; Priesnitz and Becker, 2018). Additionally, the biogenesis of iron-sulfur clusters (ISCs), cofactors that associate with proteins in the mitochondria, cytosol, and nucleus to perform diverse functions, such as respiration, protein translation, and maintenance of the genome integrity, relies on the mitochondrial membrane potential (Veatch et al., 2009). Due to its crucial role, the loss of mitochondrial membrane potential is a point of no return that activates pathways involved in targeting damaged mitochondria for clearance like the PINK/Parkin axis (Ashrafi and Schwarz, 2013). To avoid cell death, mitochondria can maintain the mitochondrial membrane potential during states of respiratory chain disruption by reversing complex V function at the expenses of glycolytic ATP. Even cells depleted of mitochondrial DNA (ρ0 cells) can maintain a mitochondrial membrane potential through the electrogenic exchange of ATP4- for ADP3- by the adenine nucleotide carrier. Importantly, this process depends on the function of an incomplete ATP synthase where the F1 component consumes glycolytic ATP (Buchet and Godinot, 1998; Appleby et al., 1999). The expression of ATPIF1, an endogenous physiological inhibitor of the F0F1-ATPase, is known to reduce the activity of the F1 component and diminish mitochondrial membrane potential in ρ0 cells and consequently its loss helps ρ0 cells sustain a mitochondrial membrane potential (Chen et al., 2014; Martínez-Reyes et al., 2016). Additional mechanisms by which cells sustain a mitochondrial membrane potential, and therefore avoid cell death when the ETC is impaired, are unknown. In this study, we addressed this question by conducting a genome wide positive selection CRISPR screen using a combination of complex I (piericidin A), complex III (antimycin A) and complex V (oligomycin) inhibitors. Our screen identified genes involved in mitochondrial protein translation and ATP synthesis as essential for the induction of cell death when cells lose their mitochondrial membrane potential.

## Materials and Methods

### Cell Culture, Cell Viability and Mitochondrial Membrane Potential Analysis

143B-CYTB-WT and 143B-CYTB-∆ cells were previously described (Weinberg et al., 2010). Cells were grown in DMEM containing 4.5 g/L D-glucose, 4 mM l-glutamine (Gibco; 11965-126) supplemented with 10% Nu-serum IV (Corning), 1 mM methyl pyruvate (Sigma), 400 μM uridine (Sigma), 1% HEPES (Gibco) and 1% antibiotic-antimycotic (Gibco) at 37°C with 5% CO_2_ and 95% humidity. To assess the percentage of dead cells, approximately 3.5 × 10^4^ cells were plated on 6-well plates. Cells were treated with 100 nM antimycin A (Sigma), 100 nM piericidin A (Sigma) and 100 nM oligomycin (Sigma) and expanded in the presence of methyl pyruvate and uridine. To assess viability, cells were stained with DAPI and analyzed by flow cytometry using BD Fortessa analyzers. The membrane potential of mitochondria was analyzed by flow cytometry using the potential dependent fluorescent dye tetramethylrhodamine (TMRE, Molecular Probes). After 3 days of treatment, cells were stained with 50 nM TMRE for 20 min at 37°C. Cells were then washed, DAPI added, and analyzed using BD Fortessa analyzers. Mitochondrial membrane potential was expressed as corrected Mean Fluorescence Intensity (MFI) values of TMRE after adding the mitochondrial uncoupler carbonyl cyanide-chlorophenylhydrazone (CCCP, 50 µM) to each sample for 10 min. Data were analyzed with the FlowJo software 10.4.2.

### CRISPR-Based Positive Selection Screen

For the positive selection screen, 140 × 10^6^ 143B-CYTB-WT cells were transduced with the Human CRISPR Knockout sgRNA Pooled Library (Brunello) from David Root and John Doench (Addgene #73178) (Doench et al., 2016). After library addition, cells were centrifuged for 50 min followed by 6 h incubation at 37°C. Infected cells were selected with puromycin (1 μg/ml) for 4 days. To set up the screen, 70 × 10^6^ cells were seeded in the presence of 100 nM antimycin A (Sigma), piericidin A (Sigma) and oligomycin (Sigma). 20 × 10^6^ cells were added to control flasks with no treatment. Treated cells were passaged for 2.5 or 6 weeks before isolating genomic DNA for sequencing. sgRNA sequences were amplified by PCR, with the addition of index and Illumina adapter sequences (P5, P7). Sequencing was done by NextSeq 2500 (Illumina). sgRNA abundance was measured and compared among the treated and untreated populations. Individual sgRNA frequencies were calculated by dividing its read number by the total number of sgRNAs reads after a pseudocount of one. The average sgRNA frequency for each gene (4 sgRNA/gene) was calculated and normalized to untreated. An excel spreadsheet is in Supplementary Material displaying sgRNA frequencies for each particular gene at 2.5 and 6 weeks of treatment with mitochondrial inhibitors. Functional enrichment analysis were conducted using STRING (Szklarczyk et al., 2019).

## Results and Discussion

**FIGURE 1:**
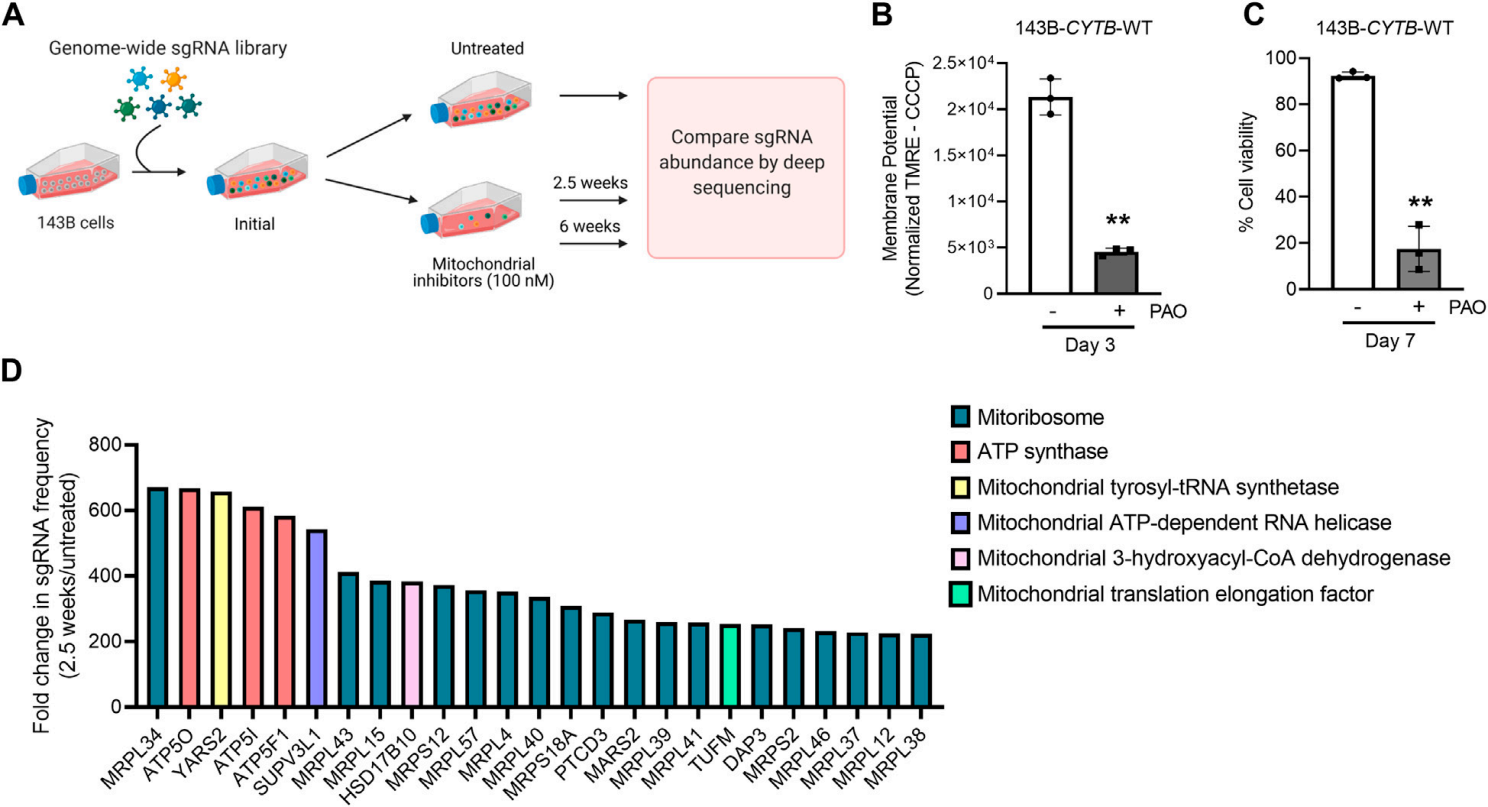
Subunits of the ATP synthase and the mitoribosome are essential to induce cell death upon mitochondrial dysfunction. **(A)** Schematic representation of the CRISPR-based positive selection screen. **(B)** Cells were treated with 100 nM pericidin A, antimycin A, and oligomycin for 3 days and mitochondrial membrane potential was measured. The value represents the MFI of TMRE corrected with the value from the same sample after CCCP treatment. **(C)** Cells were treated with 100 nM pericidin A, antimycin A, and oligomycin for 7 days and the percentage of cellular viability was assessed. **(D)** The gene scores for the top 25 genes following treatment with mitochondrial inhibitors of complexes I, III and V for 2.5 weeks. *MRPL34*, *ATP5O* and *YARS2* are the three best scoring genes. Panel 1A was created with BioRender.com.

**FIGURE 2:**
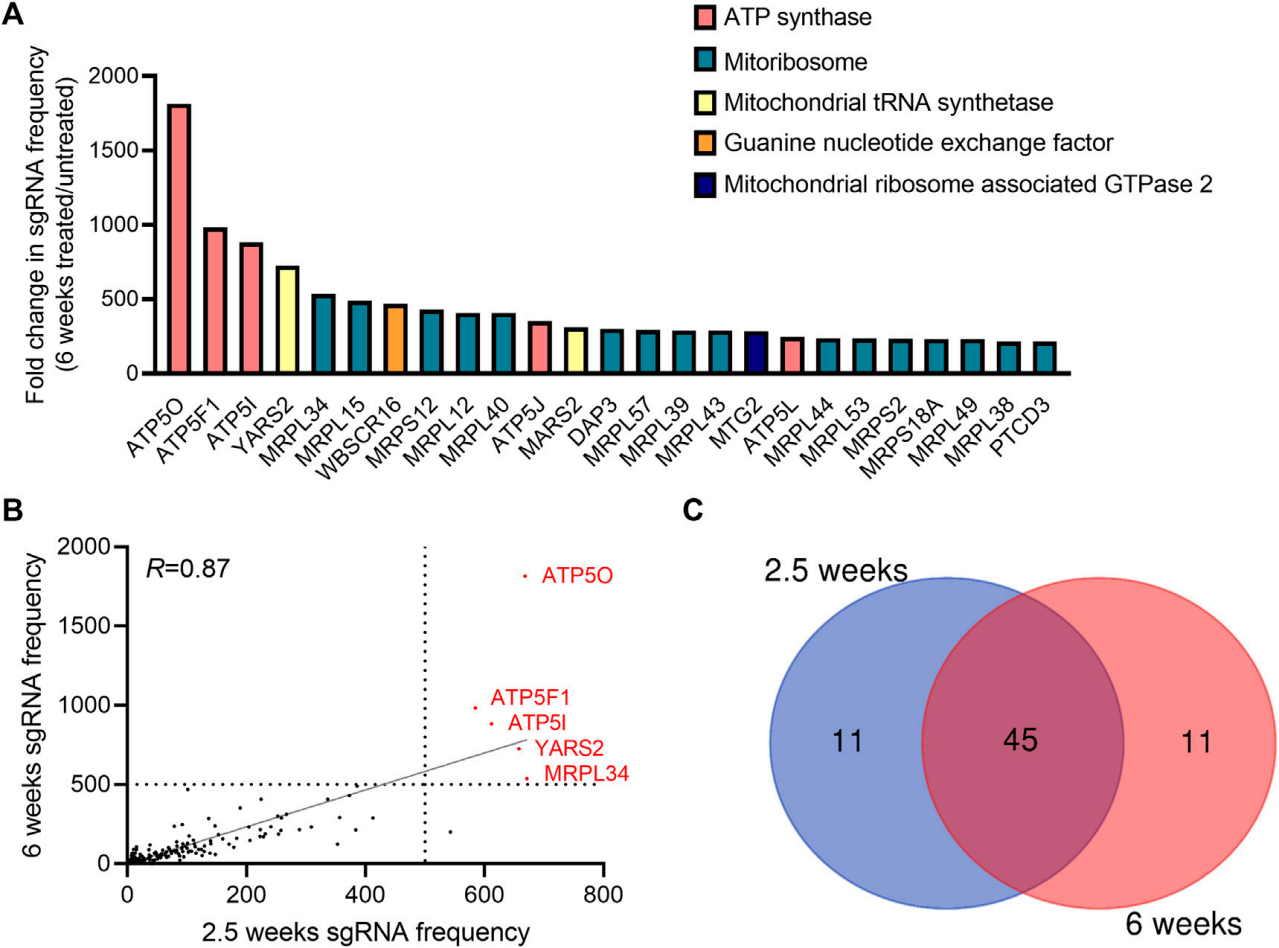
Genes essential to trigger cell death upon long-term induction of dysfunctional mitochondrial. **(A)** The gene scores for the top 25 genes following treatment with 100 nM pericidin A, antimycin A, and oligomycin for 6 weeks. Three subunits of the ATP synthase are the three best scoring genes. **(B)** A dot plot showing the post-screen frequency of sgRNAs targeting a particular gene in treated (100 nM PAO) cells for 2.5 versus 6 weeks. Results yielded the same 5 top gene hits: *ATP5O*, *ATP5F1*, *ATP5I*, *YARS2* and *MRPL34*. **(C)** Venn diagram shows the overlaps between the hits among the sgRNAs with a fold change enrichment higher than 100.

To identify genes necessary for the induction of cell death when the mitochondrial membrane potential is lost, we conducted a CRISPR-Cas9 based positive-selection screen for genes whose loss allowed cell survival in the presence of 100 nM of complex I (piericidin A), complex III (antimycin A) and complex V (oligomycin) inhibitors (Figure 1A). Disruption of the ETC with this concentration of mitochondrial inhibitors in 143B-CYTB-WT cells decreased mitochondrial membrane potential (Figure 1B) after 3 days of treatment without affecting cell viability (Supplementary Figure S1A). Complex III deficient cells (143B-CYTB-∆) are dependent on an intact complex V to maintain the mitochondrial membrane potential by reversing its function. Consequently, we observed a decrease in the mitochondrial membrane potential of 143B-CYTB-∆ when treated with oligomycin (Supplementary Figure S1B). After 3 days of treatment, the cell viability of 143B-CYTB-∆ cells treated with oligomycin remained unaffected (Supplementary Figure S1C). The longer exposure of 143B-CYTB-WT cells to the inhibitors (7 days) was sufficient to induce cell death (Figure 1C). 143B-CYTB-WT cells were transduced with a genome wide lentiviral single-guide RNA (sgRNA) library containing 4 unique sgRNAs for each gene (76,441 sgRNAs total) (Doench et al., 2016). The knockout pool of cells were then cultured in the presence or absence of 100 nM mitochondrial inhibitors (piericidin, antimycin and oligomycin, hereafter referred to as PAO) for 2.5 weeks. A pool of cells treated with PAO was also maintained for 6 weeks. Following this time, genomic DNA was isolated from the untreated and treated cell populations and deep sequencing was performed to compare the abundance of each sgRNA. The change in abundance of each sgRNA was assessed as the average frequency of individual sgRNA targeting each gene (4 sgRNA/gene) in the PAO-treated population normalized to untreated. If an sgRNA knocked out a gene that is essential for the induction of cell death when the mitochondrial membrane potential is lost, cell viability would be unaffected by selection with 100 nM PAO. The sgRNAs for those genes would be enriched in the treated surviving population, giving these genes a positive differential gene score compared to the untreated population (Figure 1D; Supplementary Table S1). Our second top gene hit was ATP5O, which is the oligomycin sensitivity conferring protein in the ATP synthase, a positive control that validated the robustness of the screen. Two other subunits of the ATP synthase, ATP5I and ATP5F1, were among the top 5 hits. In total, disrupting 9 of the 20 subunits of mitochondrial ATP synthase gave cells a survival advantage during PAO treatment. We have previously demonstrated that KO of ATPIF1 reverses the drop in membrane potential and increases cell proliferation when mitochondrial function is disrupted (Martínez-Reyes et al., 2016). As an additional positive control, the four gRNAs targeting ATPIF1 were found to be enriched (Supplementary Figure S1D). The gene with the highest score was MRPL34, a subunit of the mitoribosome. A vast majority of mitoribosomes subunits were enriched under PAO treatment conditions. Among the genes whose loss conferred resistance to mitochondrial loss of membrane potential, the tyrosyl-tRNA synthetase YARS2 had the third highest gene score (Figure 1D). About half of the mitochondrial tRNA ligases were found to be enriched. Since the treatment is what drives the selective pressure on the population of sgRNA infected cells, we decided to maintain a pool of cells treated for an extended time to increase our confident on the results. Importantly, after 6 weeks of treatment, results yielded the same top 5 gene hits (Figures 2A,B). Following the same trend as the treatment for 2.5 weeks, loss of subunits of the ATP synthase and the mitoribosome conferred a survival advantage to cells with disrupted mitochondrial ETC and represented the majority of the 25 gene hits (Figure 2A). From the genes with a fold change higher than 100, 45 were the same at 2.5 and 6 weeks (Figure 2C). A functional enrichment analysis of results after 2.5 weeks of PAO treatment showed that the pathways enriched are mainly part of biological processes associated with mitochondrial protein translation (Supplementary Table S2). Similar enriched pathways were found after 6 weeks of PAO treatment (Supplementary Table S3). It should be noted that based on the STRING algorithm, heavily up-regulated genes in the pathway can drive the enrichment of the whole pathway. This leads to cases where genes not enriched in our data set, but associated with enriched pathways are listed in Supplementary Tables S2, S3. Overall, our results indicate that ATP synthase and mitochondrial translation are required for cell death after loss of mitochondrial membrane potential.

The maintenance of the mitochondrial membrane potential is a key function of the ETC and is essential for mitochondrial homeostasis. Loss of the mitochondrial membrane potential triggers mechanisms to selectively eliminate damaged mitochondria and apoptosis (Gottlieb et al., 2003). Beyond its requirement for ATP synthesis, the membrane potential is necessary to support many other essential biological processes, including mitochondrial biogenesis and the transport of biosynthetic intermediates between the mitochondrial matrix and the cytoplasm. In cancer cells, we have recently uncovered that genetic engineering of the ETC to maintain the TCA cycle function in the absence of mitochondrial ATP production is sufficient to support tumor growth (Martínez-Reyes et al., 2020). This indicates that glycolytic derived ATP is sufficient to sustain tumor growth. Other functions of mitochondria that depend on the maintenance of the membrane potential and that are mostly related to cell signaling might be more important in certain contexts. For example, under hypoxia, a membrane potential is necessary to release ROS which is required for the stabilization of HIF1α (Chandel et al., 1998; Martínez-Reyes et al., 2016). Reminiscent of its endosymbiotic origin, mitochondria contain their own genome that encodes for a small number of proteins, while the vast majority of mitochondrial proteins are produced on cytosolic ribosomes and further imported into the mitochondria. The formation of respiratory chain complexes for instance depends on the coordinated biogenesis of mitochondrial encoded and nuclear-encoded subunits (Couvillion et al., 2016). An essential function supported by the mitochondrial membrane potential is the import of proteins encoded by the nuclear genome. The mitochondrial proteins translation machinery itself requires the import of mitorribosomal subunits to the organelle. Our results indicate that under the absence of a mitochondrial membrane potential, inhibiting the translation of mitochondrial proteins is a survival strategy. The exact mechanism that mediates cell survival when proteins related to mitochondrial translation are lost requires further investigation. The results of our screen are intended to provide a valuable resource of potential targets that might confer resistance to the loss of mitochondrial membrane potential. Although further validation is required, the following reasons indicate the potential relevance of our hits. Several genes from the same cellular structure, mitoribosomes, or the H + -ATP synthase are among the top hits of our screens both at 2.5 weeks and at 6 weeks. These results increased our confidence in the relevance of the pathways involved as essential for the induction of cell death when cells lose their mitochondrial membrane potential. Both time points, 2.5 and 6 weeks yield the same top hits and gRNAs targeting ATPIF1, a gene previously known to be important for Preventing the maintenance of the mitochondrial membrane potential when the electron transport chain is disrupted, were found enriched. Moving forward, it will be interesting to study whether in diseases that often display dysfunctional mitochondria, such as neurodegeneration, targeting these pathways will have a beneficial effect.

## Data Availability

The original contributions presented in the study are included in the article/Supplementary Material, further inquiries can be directed to the corresponding author.

## Supplementary Material

Supplementary Figure 1
**(A)** 143B-CYTB-WT cells were treated with 100 nM pericidin A, antimycin A, and oligomycin for 3 days and the percentage of cellular viability was assessed. **(B,C)**. 143B-CYTB-∆ cells were treated with 100 nM oligomycin for 3 days and mitochondrial membrane potential **(B)** and cell viability **(C)** were assessed. **(D)**. Post-positive selection screen changes in the abundance of individual sgRNAs targeting ATPIF1 in the presence of PAO during 2.5 and 6 weeks. Individual sgRNA frequencies were calculated by dividing its read number by the total number of sgRNAs reads after a pseudocount of one. The value of each sgRNA at 2.5 and 6 weeks was normalized to the average value of sgRNAs in the untreated control.

Supplementary Table 1 A list with the differential gene scores of all genes covered by our screen.

Supplementary Table 2 A complete list of genes associated with the functional enriched pathways after 2.5 weeks of treatment. Genes within the bottom direction have a positive log value which means that the sgRNAs targeting that particular gene were found enriched.

Supplementary Table 3 List of enriched pathways including the genes associated with them after 6 weeks of treatment. Genes within the bottom direction have a positive log value which means that the sgRNAs targeting that particular gene were found enriched.
