# Supplementary figures and images for: Genes Involved in Maintaining Mitochondrial Membrane Potential Upon Electron Transport Chain Disruption
Source: Front Cell Dev Biol. 2022 Feb 16;10:781558. doi: 10.3389/fcell.2022.781558 (PMC8888678; doi:10.3389/fcell.2022.781558)

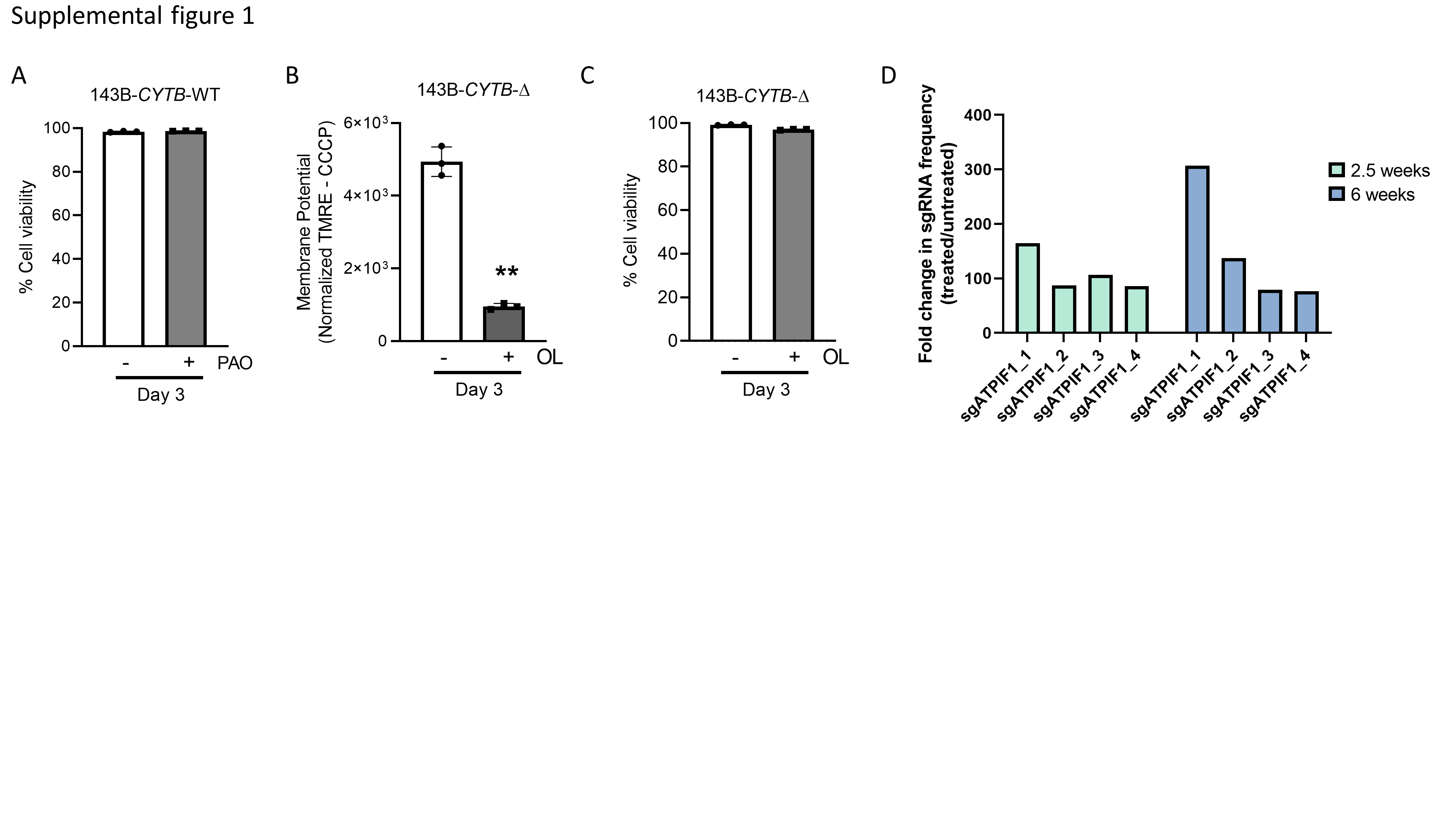

Supplement: Supplementary file 3 [file Image1.tif]
